# Using Intervention Mapping to Develop and Adapt Two Educational Interventions for Parents to Increase HPV Vaccination Among Hispanic Adolescents

**DOI:** 10.3389/fpubh.2018.00164

**Published:** 2018-06-15

**Authors:** Serena A. Rodriguez, Angelica M. Roncancio, Lara S. Savas, Diana M. Lopez, Sally W. Vernon, Maria E. Fernandez

**Affiliations:** ^1^Center for Health Promotion and Prevention Research, School of Public Health, The University of Texas Health Science Center at Houston, Houston, TX, United States; ^2^Department of Clinical Sciences, University of Texas Southwestern Medical Center, Dallas, TX, United States

**Keywords:** HPV vaccination, Hispanic adolescents, intervention development, intervention adaptation, Intervention Mapping

## Abstract

**Introduction:** Effective interventions to increase HPV vaccination are needed to reach national vaccination goals and to reduce later HPV-related cancer disparities. We used Intervention Mapping (IM) to develop and adapt a theory- and evidence-based educational intervention targeting parents of Hispanic adolescents to increase HPV vaccination.

**Methods:** We followed IM steps 1–6 to: (1) develop a logic model and identify modifiable factors associated with vaccination among Hispanic adolescents by conducting literature reviews, focus groups, and in-depth interviews with Hispanic parents; (2) develop outcomes, write performance objectives, and develop a matrix of change objectives; (3) develop and identify a program theme, program components, theoretical methods, and practical applications; (4) develop an intervention design plan; (5) develop implementation strategies; and (6) develop an evaluation plan. We completed Steps 1–6 for to develop an intervention targeting parents of females, and we followed the steps again to adapt the program once HPV vaccine recommendations included males.

**Results:** The program *Por Nuestras Hijas* (For Our Daughters) included two components: a print *fotonovela* and a tailored interactive multimedia intervention (TIMI). The program utilized the methods tailoring, targeting, framing, anticipated regret, modeling, skill building, and education and counseling to target the following determinants: parental knowledge, attitudes, self-efficacy, skills, perceived benefits/barriers, perceived susceptibility, perceived norms, and outcome expectations as modifiable factors influencing HPV vaccination. Lay health workers implemented the program in community clinics. A logic model of change guided evaluation planning. We later adapted the outcome and intervention content for parents of Hispanic adolescent males and changed the theme to *Por Nuestros Hijos* (For Our Children). Throughout the development and adaptation processes, we relied on theory, empirical evidence, and new data to make decisions.

**Discussion:** IM provided a systematic methodology for program development and adaptation. Tasks in each step built upon one another integrating findings from the literature, previous research, qualitative findings, and theory to develop two educational programs for parents to increase HPV vaccination. The systematic process allowed us to develop messages and materials targeting factors beyond HPV knowledge or awareness to create behavior change.

## Introduction

Persistent human papillomavirus (HPV) infection can lead to anogenital cancers, oropharyngeal cancer, and genital warts (1). In 2017, cervical cancer was the most prevalent HPV-related cancer with nearly 12,000 new cases per year in the United States (2). Cervical cancer disproportionality affects Hispanic women who have higher cervical cancer rates than their non-Hispanic counterparts and who have the second highest cervical cancer mortality rate after Black women (3, 4). Hispanic women also have lower cervical cancer screening rates than non-Hispanic women contributing to cervical cancer morbidity and mortality disparities (5).

The HPV vaccine can protect against the types of HPV that can lead to cervical cancer, other anogenital cancers, oropharyngeal cancer, and genital warts in both men and women. The Centers for Disease Control Advisory Committee for Immunization Practices (ACIP) recommends providers administer the HPV vaccine to adolescent males and females at ages 11–12 (6). While HPV vaccination rates in the United States have improved incrementally over the last several years, they remain below national benchmarks (7). Specifically among Hispanic adolescents, 68% of females aged 13–17 years, and 59% of males aged 13–17 years initiated the vaccine in 2015. However, only 46% of females and 35% of males completed the vaccine series (8). Theory- and evidence-based HPV vaccination interventions aimed at increasing series initiation and completion by targeting the multiple factors influencing vaccination rates are needed for Hispanic adolescent populations to reduce later cancer disparities (5, 9).

Theory- and evidence-based interventions have been shown to be more effective than non-theory based interventions (10). Intervention Mapping (IM) is a systematic framework for developing, implementing, and adapting theory- and evidence-based interventions (11). Cancer control and prevention researchers and program planners have used IM to develop interventions focused on sun safety and skin cancer prevention (12), smoking cessation (13) cervical cancer and breast cancer screening (14–19), and colorectal cancer screening (20). IM also provides a framework for developing implementation strategies for the adoption and implementation of interventions (21) and clinical guidelines (22) and for systematically adapting existing evidence-based programs (23).

This paper describes the use of IM to develop and adapt two interventions for parents aimed at increasing HPV vaccine uptake among Hispanic adolescents. These interventions were developed as part of a larger comparative effectiveness study to develop and evaluate a print vs. tailored intervention delivered by lay health workers in clinic settings. Initially, we followed IM to develop interventions focused exclusively on parents of Hispanic female adolescents to increase HPV vaccination among this population. The interventions, packaged as *Por Nuestras Hijas* (For Our Daughters), included a print *fotonovela* and a tailored interactive multimedia intervention (TIMI) delivered as an application on a tablet. *Fotonovela*s are brief print stories with pictures and dialog popular among Hispanic populations in conveying health information. Once recommendations changed and included HPV vaccination for boys, we used IM to adapt the interventions, repackaged as *Por Nuestros Hijos* (For Our Children).

## Methods

**Figure 1:**
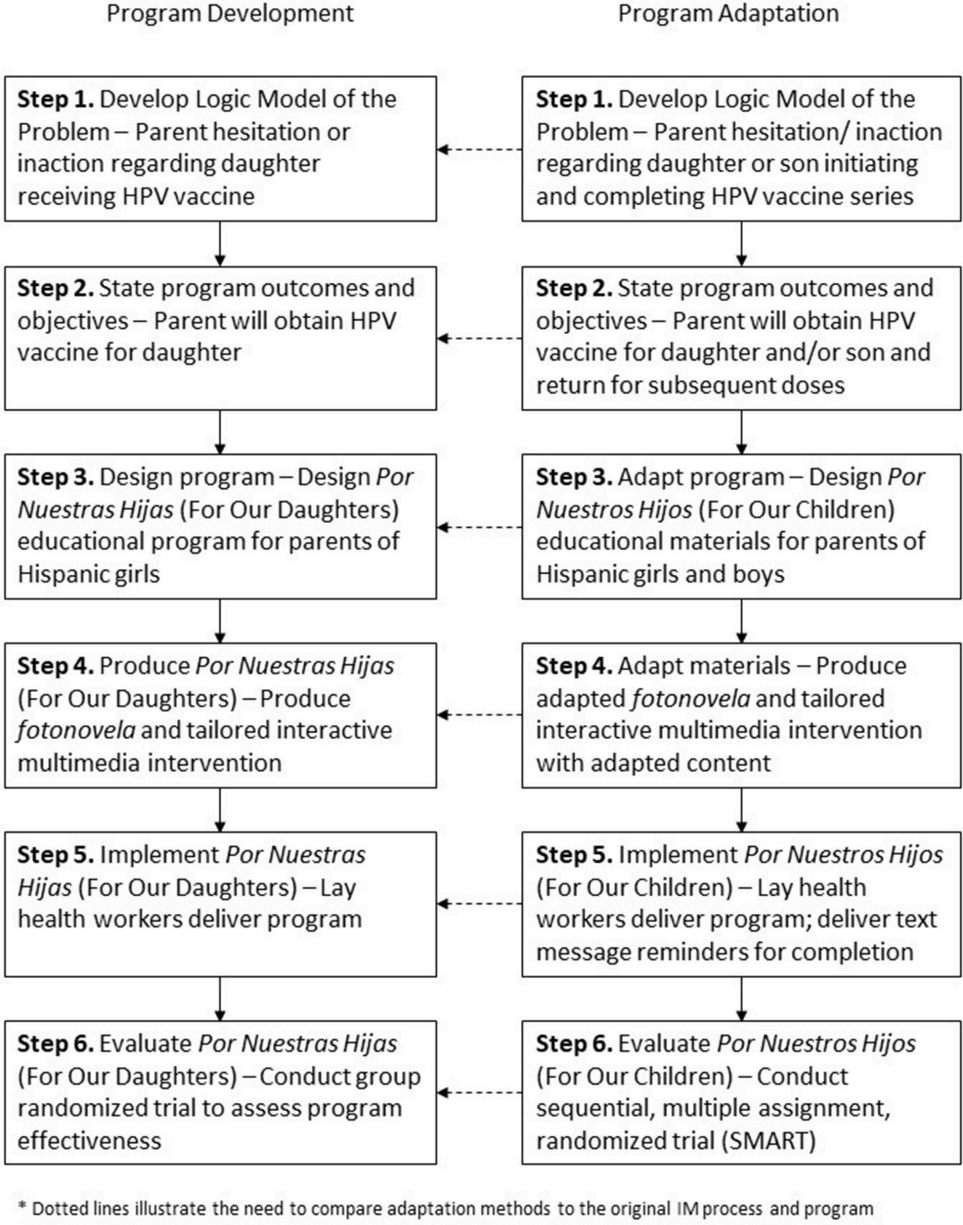
PNH development and adaptation process.

Intervention Mapping guides program planners through six steps from needs assessment to program evaluation. The steps include: (1) developing a logic model of the problem, (2) identifying program outcomes and objectives, (3) designing the program, (4) producing the program, (5) planning for program implementation, and (6) planning for evaluation (11). Below we detail IM Steps 1–6 methods to develop and adapt two educational interventions for Hispanic parents of adolescents aged 11–17 years (Figure 1). While we briefly described the IM Step 6 in terms of evaluation planning, evaluation outcomes are not described as the effectiveness trial is currently ongoing. The University of Texas Health Science Center at Houston Institutional Review Board approved all research conducted during program development and adaptation.

### *Por Nuestras Hijas* program development

#### Step 1. Develop a logic model of the problem

The purpose of IM Step 1 is to conduct a needs assessment and develop a logic model of the problem (parent hesitation or inaction regarding adolescent child's HPV vaccination). To inform our needs assessment and logic model, we identified the following: (1) the burden of cervical cancer among low income or underinsured Hispanic women; (2) rates of HPV vaccination among low income and underinsured Hispanic female adolescents; (3) Hispanic parental beliefs, barriers, or attitudes toward the HPV vaccine amon; (4) and parental decision-making processes for obtaining the vaccine for their daughters.

IM recommends first forming a planning group made up of community stakeholders, members of the target population, and potential program implementers who work collaboratively with intervention developers to identify health problems and work together to develop solutions (11). Our planning group consisted of academic researchers and intervention planners, community leaders, and a prominent *promotora* (health worker) association to help guide program development, implementation, and adaptation decisions.

The team conducted a literature review to identify factors related to parental acceptance of the HPV vaccine and parental beliefs and attitudes related to HPV vaccine decision-making. A medical librarian skilled in designing systematic reviews developed our search, which included MeSH terms related to HPV vaccination, parent decision-making and vaccinations, parental acceptance of the HPV vaccine, and parental beliefs and attitudes toward the HPV vaccine for their daughters. Studies conducted in the United States between 2001 and 2010 published in English were included in the search. Studies were not limited to those with Hispanic participants only.

Finally, focus groups and in-depth interviews were conducted with Hispanic parents of adolescent females aged 11–17 years to further understand parental acceptance of the HPV vaccine for their daughters and beliefs and attitudes related to the vaccination. Research staff placed recruitment flyers in local community centers and clinics serving predominantly Hispanic populations. Parent eligibility requirements included: (1) identify as Hispanic/Latina/o; and (2) report having a daughter aged 11–17 years. Bilingual facilitators trained in qualitative research methods conducted a total of four focus groups and two in-depth interviews. All focus groups and interviews were recorded and later transcribed for analysis. All participants completed informed consent documents prior to the focus group or interview, and all received a $25 gift card for participating.

#### Step 2. Identify program outcomes and objectives—logic model of change

The purpose of IM Step 2, is to state program outcomes and objectives and develop a matrix of change objectives to guide program development. During this step, we stated the overall behavioral outcome to be accomplished as a result of our intervention and developed a detailed list of performance objectives, or sub-behaviors needed in order to achieve the outcome (11).

To construct the matrix of change objectives, performance objectives were included as row headings and modifiable factors positively associated with vaccine decision-making (determinants) as column headings. IM Step 1 findings informed the selection of determinants for the matrix. We crossed performance objectives and determinants to produce change objectives. Change objectives describe what must change in the determinant in order for an individual to perform the performance objective (11). The matrix of change objectives served as a blueprint for further program development and guided the selection of intervention methods, practical applications, and messages. Step 2 elements were organized into a logic model of change illustrating the expected change mechanisms leading to the desired behavior—HPV vaccine uptake.

#### Step 3. Design *Por Nuestras Hijas*

The purpose of IM Step 3 is to develop a program theme, identify program components, map change objectives and determinants to theoretical methods, and develop practical applications to operationalize methods. Our team collaborated with the planning group to develop a theme for the intervention, identify program components, decide on tangible products for parents, and discuss how parents would interact with the intervention. Guided by tables of methods to address specific determinant in the Intervention Mapping text (11), the group selected theoretical methods to influence specific determinants. Theoretical methods are techniques for influencing determinants to ultimately create behavior change (11). We then developed practical applications, or the specific ways we would operationalize the theoretical methods. The setting (clinics), feasibility of delivery, literacy level of parents, and preferences for educational material were considered throughout this process.

#### Step 4. Produce *Por Nuestras Hijas*

For both program development and adaptation, a production plan guided the creation and adaptation of materials. The plan included detailed flowcharts, developed mock-ups, created content, and produced all materials. To produce materials, collaborators included an application design team, a graphic designer, a talent consultant and agency, a video production team, actors, video editors, and a photographer.

#### Step 5. Implement *Por nuestras hijas*

In IM Step 5 for both program development and adaptation, we developed implementation strategies to build lay health worker capacity to deliver the program. To do so, we completed IM Steps 1–4 again with lay health workers in mind. This included identifying specific steps lay health workers would have to do in order to implement the interventions, determinants that may influence lay health workers' abilities to implement, theoretical methods to influence those determinants, and materials needed to deliver the interventions.

#### Step 6. Evaluate *Por Nuestras Hijas*

As a first evaluation step in both program development and adaptation, we validated that program content was consistent with the program plan by comparing content with the matrix of change. Creating a table with all determinants, change objectives, theoretical methods, and practical applications enabled us to assess whether the interventions addressed each change objective.

Next, we designed a comparative effectiveness study to assess the intervention effect on increasing vaccination uptake among Hispanic adolescents. Previous IM Steps informed the evaluation in several ways. First, the matrix of change objectives guided measurement development since they clearly describe the specific changes expected because of the program. The logic model guided development of indicators and measures to identify predictors, mediators, and moderators of HPV vaccination. The logic model of change also guided the evaluation of program implementation and informed our process evaluation. A complete description of the group randomized controlled trial including results is forthcoming. However, in short, thirty federally qualified health centers and community clinics to participate in the study. Lay health workers recruited participants from within the clinics and followed protocols to assess eligibility, obtain informed consent, administer surveys, and implement the interventions.

### *Por Nuestros Hijos* program adaptation

#### Step 1. Develop a logic model of the problem

During program adaptation Step 1, the aim was to identify factors associated with parental vaccine decision-making for males that may differ from those identified for parents of females. If significant differences existed, the program would likely require more extensive adaptations. Similar to methods in Step 1 for program development, we described the disease burden among the target population, parental barriers to obtaining the HPV vaccine for their sons, and the parental decision-making process for vaccinating sons.

An updated literature search was conducted in PubMed for studies assessing the following: (1) correlates of HPV vaccination in boys; (2) parents' acceptance or intention to vaccinate their sons against HPV; (3) parents' attitudes, knowledge, and acceptability of the HPV vaccine for male adolescents; and (4) barriers to HPV vaccination for male adolescents. The search was limited to US populations and to studies published in English through January 2015.

Step 1 also included three focus groups and five individual interviews with 20 Hispanic parents of males ages 11–17 recruited from local community centers and clinics. The purpose was to understand parental beliefs about the HPV vaccine for males and to understand parental decision-making to vaccinate their sons. Trained bilingual facilitators conducted the focus groups and interviews, which lasted between 60 and 85 min each. All sessions were conducted in Spanish. Facilitators obtained informed consent from each participant prior to each session, and all sessions were recorded and transcribed for analysis. Parents received $20 gift cards for participating.

#### Step 2. Identify program outcomes and objectives—logic model of change

Throughout the adaptation process, we considered whether the adapted intervention needed a new behavioral outcome and whether steps parents took to obtain the HPV vaccine for sons different from steps for obtaining the vaccine for daughters. All steps outlined above were followed to state the adapted behavioral outcome, identify adapted performance objectives, and create an adapted matrix of change objectives.

#### Step 3. Design *Por Nuestros Hijos*

During program adaptation, we reexamined the program theme, components, and theoretical methods, and practical applications to assess applicability to our new population. To ensure all content was relevant, we compared change objectives from the matrices for males to the materials previously developed for females. For example, if a change objective stated specific knowledge a parent should have, all materials were checked for that content. For all change objectives not addressed in the materials, we created new content.

## Results

### For our daughters program development

#### Step 1. Develop a logic model of the problem

The literature search resulted in 30 studies examining factors associated with HPV vaccination among adolescent females. Modifiable factors associated with HPV vaccination included knowledge, attitudes toward the HPV vaccine, self-efficacy and communication skills, perceived benefits of the vaccine, perceived barriers to vaccination, HPV risk perception, and concerns about HPV vaccine safety (24–26). Non-modifiable factors included income, parental history of sexually transmitted infections, mother's Pap testing history, and parental education level (25, 27–29). The literature review also elucidated information sources where parents learned about HPV and the HPV vaccine including pamphlets, brochures, the internet, television media, and health care providers (30).

Findings from the focus groups and interviews confirmed that the modifiable factors identified in the literature search were applicable to our population. For example, Hispanic parents had low knowledge and awareness about HPV and the HPV vaccine, and parents expressed concerns about vaccine safety and side effects. Importantly, parents wanted information on how to speak with their daughter's provider about HPV and the HPV vaccine.

**Figure 2:**
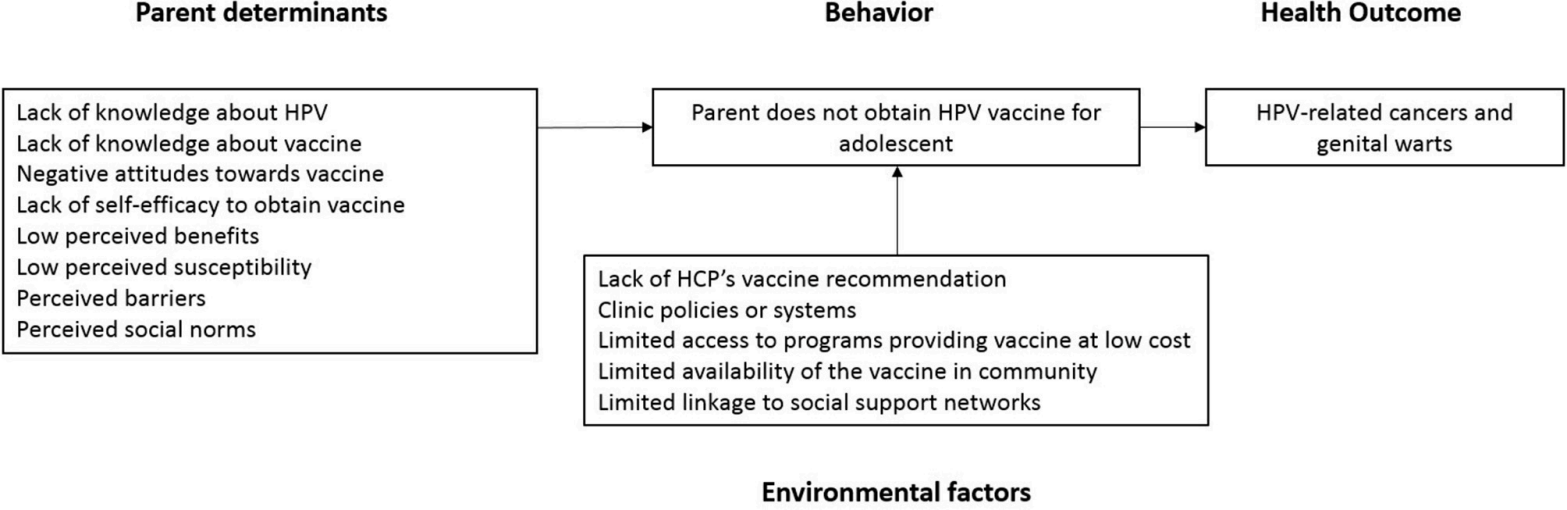
Logic model of the problem.

Our complete logic model of the problem included the behavior of interest (lack of HPV vaccination), the related health consequences (HPV-related cancers and genital warts), and determinants, or parental factors associated with the decision not to vaccinate (Figure 2).

#### Step 2. Identify program outcomes and objectives—logic model of change

The overall behavioral outcome focused on parents: Parent will obtain the HPV vaccine for daughter. We identified five performance objectives associated with the outcome (Table 1). Determinants to increase vaccination behaviors identified in Step 1 included knowledge, attitudes toward the HPV vaccine, self-efficacy and communication skills, perceived benefits of the vaccine, perceived barriers to vaccination, HPV risk perception, and concerns about HPV vaccine safety. The matrix of change objectives described the changes in determinants needed for each specified performance objective (Table 2).

**Table 1:** Behavioral outcome with associated performance objectives.

**Behavioral Outcome: Parent obtains the HPV vaccine for daughter**
**Performance Objectives:** (1) Parent considers and decides to vaccinate daughter against HPV (2) Parent discusses HPV vaccine with health care provider
a. Parent tells provider he/she is considering the HPV vaccine for daughter b. Parent asks the provider questions or communicates concerns, if any c. Parent tells provider he/she wants daughter to receive the HPV vaccine
(3) Parent identifies payment mechanism for vaccine (4) Parent obtains the first dose for daughter (5) Parent ensures daughter receives next two vaccine doses
a. Parent makes follow-up appointments b. Parent writes down next doses schedules c. Parent and daughters attend appointments

**Table 2:** Intervention mapping partial matrix for the behavioral outcome “parent obtains the HPV vaccine for daughter.”

**Performance objectives**	**Determinants**							
***Parent will:***	**Knowledge**	**Attitude**	**Self-efficacy**	**Skills**	**Perceived benefits/barriers**	**Perceived susceptibility**	**Perceived social norms**	**Outcome expectations**
PO1. Consider and decide to vaccinate daughter against HPV.	K.1.a. Recognize HPV is the most common sexually transmitted infection and can lead to cancer. K.1.b. Understand the link between HPV and cervical cancer. K.1.c. Recognize that there is an HPV vaccine to protect against HPV-related cancers. K.1.d. Recognize that daughter is eligible to receive the HPV vaccine. K.1.e. Understand that the vaccine is recommended for girls before sexual debut.	A.1.a. Believe that the HPV vaccine is important for daughters. A.1.b. Believe the HPV vaccine to be safe for their daughters. A.1.c. Believe that HPV infection can have serious consequences. A.1.d. Believe that the vaccine will not encourage sexual activity. A.1.e. Believe that not vaccinating will result in feelings of guilt and regret if daughter later develops cervical cancer.	SE.1.a. Demonstrate confidence in ability to process information about the HPV vaccine. SE.1.b. Demonstrate confidence in ability to decide whether or not to vaccinate daughter.	S.1.a.Express ability to process information about the HPV vaccine. S.1.b. Express ability to decide whether or not to vaccinate daughter.	Pbe.1.a. Recognize that the HPV vaccine can prevent HPV, cervical cancer, and HPV-related cancers. Pba.1.b. Understand that the benefits of HPV vaccination are greater than the barriers.	PS.1.a. Recognize HPV is the most common sexually transmitted infection, and daughter is susceptible once sexually active. PS.1.b. Recognize that daughter will be at risk for HPV and cervical cancer if not vaccinated.	SN.1. Believe that other parents are vaccinating their daughters against HPV.	OE.1.a. Expect that the HPV vaccine will not produce negative results for daughters. OE.1.b. Expect the HPV vaccine can prevent cervical cancer and HPV. OE.1.c. Expect that the vaccine will not encourage sexual activity.
PO2. Discuss the HPV vaccine with health care provider (HCP). PO2.a. Tell HCP that they are considering the HPV vaccine for daughter. PO2.b. Ask HCP questions or concerns about their daughter receiving the HPV vaccine. PO2.c. Tell HCP that they want their daughter to receive the HPV vaccine.	K.2. Understand that they may have to initiate a conversation about the HPV vaccine with daughter's HCP	A.2.a. Believe that HCP will take time to discuss concerns about the HPV vaccine. A.2.b. View that discussing the HPV vaccine with HCP will address concerns. A.2.c. Believe that they have the right to initiate the discussion and ask questions.	SE.2.a. Feel confident that they can initiate a discussion even if it is not mentioned. SE.2.b. Feel confident that they can talk to HCP. SE.2.c. Feel confident about asking questions regarding the vaccine. SE.2.d. Feel confident about asking for their daughter to receive the HPV vaccine.	S.2.a. Demonstrate ability to initiate a discussion about the HPV vaccine with HCP. S.2.b. Demonstrate ability to communicate, listen and analyze the information that HCP will give them. S.2.c. Demonstrate ability to request the HPV vaccine for daughter.	Pba.2.a. Overcome feelings of fear and discomfort in order to initiate discussion with HCP about the HPV vaccine. Pbe.2.b. Believe that requesting the HPV vaccine from daughter's HCP will help daughter get vaccinated.	PS.2. Believe that their daughter could be at risk of HPV infection at some point in her life.	SN.2.a. Believe that other parents are discussing the HPV vaccine with their daughter's HCP. SN.2.b. Believe that other parents are requesting the HPV vaccine from their daughter's HCP.	OE.2.a. Expect that the HCP wants to discuss concerns. OE.2.b. Expect that discussing the HPV vaccine with HCP will address concerns.

#### Step 3. Design *Por Nuestras Hijas*

##### Program theme

The team developed the theme “*Por Nuestras Hijas* (For Our Daughters)” to convey a message of protection. Throughout the program, the theme was interwoven in two ways: (1) highlight that the HPV vaccine protects daughters from the human papillomavirus; and (2) highlight that parents protect their daughters from cancer by obtaining the HPV vaccine for them. The intervention follows the story of a mother gathering information about the HPV vaccine and making the decision to vaccinate her daughter to protect her against cancer. This protective framework is effective for this population. Messages that specifically frame the HPV virus as a threat to daughters and mothers as protectors increase Hispanic mothers' intentions to vaccinate their daughters (31).

##### Program components

Based on our previous work (32), we developed two program components: a print *fotonovela* and a tailored interactive multimedia intervention (TIMI) delivered on a tablet. The two components conveyed the story of the mother in both video and print format. Print *fotonovela*s are illustrated brief stories with pictures and dialog accompanying the images. *Fotonovela*s are soap-opera style stories, popular in Spanish-speaking societies and are often used to describe and educate Hispanic audiences about health topics. They often employ theory and evidence-based methods such as social modeling and vicarious learning, and have been shown to be effective in helping individuals personalize health issues, identify with and internalize the information being presented, and engage in positive health behaviors (33, 34).

##### Theoretical methods

Tailoring and targeting, modeling, skill building, and education and counseling were identified as key theoretical methods to address determinants influencing Hispanic parents' decision-making regarding the HPV vaccine. Tailoring presents messages or interventions based on characteristics unique to an individual. Targeting is broader and involves developing messages and interventions for a subpopulation or group with shared characteristics (35, 36). These methods were operationalized as practical applications in multiple ways. First, program components were presented in English and Spanish and used actors resembling the audience (see IM Step 4). Next, self-tailoring and automatic-tailoring pause points were included throughout the TIMI. At self-tailoring points, parents chose information they wanted to see. For example, one pause point listed common questions parents have about the HPV vaccine. Parents were able to choose all of the questions they had, and the program tailored the subsequent content accordingly. Automatic-tailoring included pause points where the program asked questions based on specific determinants. Depending on the response, the application showed different messages.

We also incorporated framing and anticipatory regret in the intervention. Gain-framing and loss-framing may be used to emphasize the advantages or disadvantages of performing a behavior, such as vaccination (37). Using loss framing, we emphasized the negative consequences of not vaccinating to elicit anticipatory regret. For low-frequency behaviors such as HPV vaccination, loss-framed messages have been found to be associated with greater behavioral intentions (38). These framing methods targeted perceived susceptibility, perceived vaccine efficacy, and behavioral intentions.

Modeling, a method from the Social Cognitive Theory (39), targeted multiple determinants such as self-efficacy and skills, and reinforced desired behaviors outlined in the performance objectives. As stated above, the theme following the story of a mother as she decides to vaccinate her daughter. Scenes in the program showed the mother modeling behaviors aligned with the performance objectives that we wanted parents to emulate.

Additional methods included education and counseling and skill building as we aimed to educate parents about the virus, the HPV vaccine, and how to request the vaccine for their adolescents. By identifying participant-specific barriers to initiation and completion through tailoring, the program provided tools and information necessary to build skills and overcome those barriers. For example, participants had the opportunity to write down questions they had for their provider after viewing content. Additionally, lay health workers clarified information that was unclear to participants and provided additional sources of information as needed.

#### Step 4. Produce *Por Nuestras Hijas*

**Figure 3:**
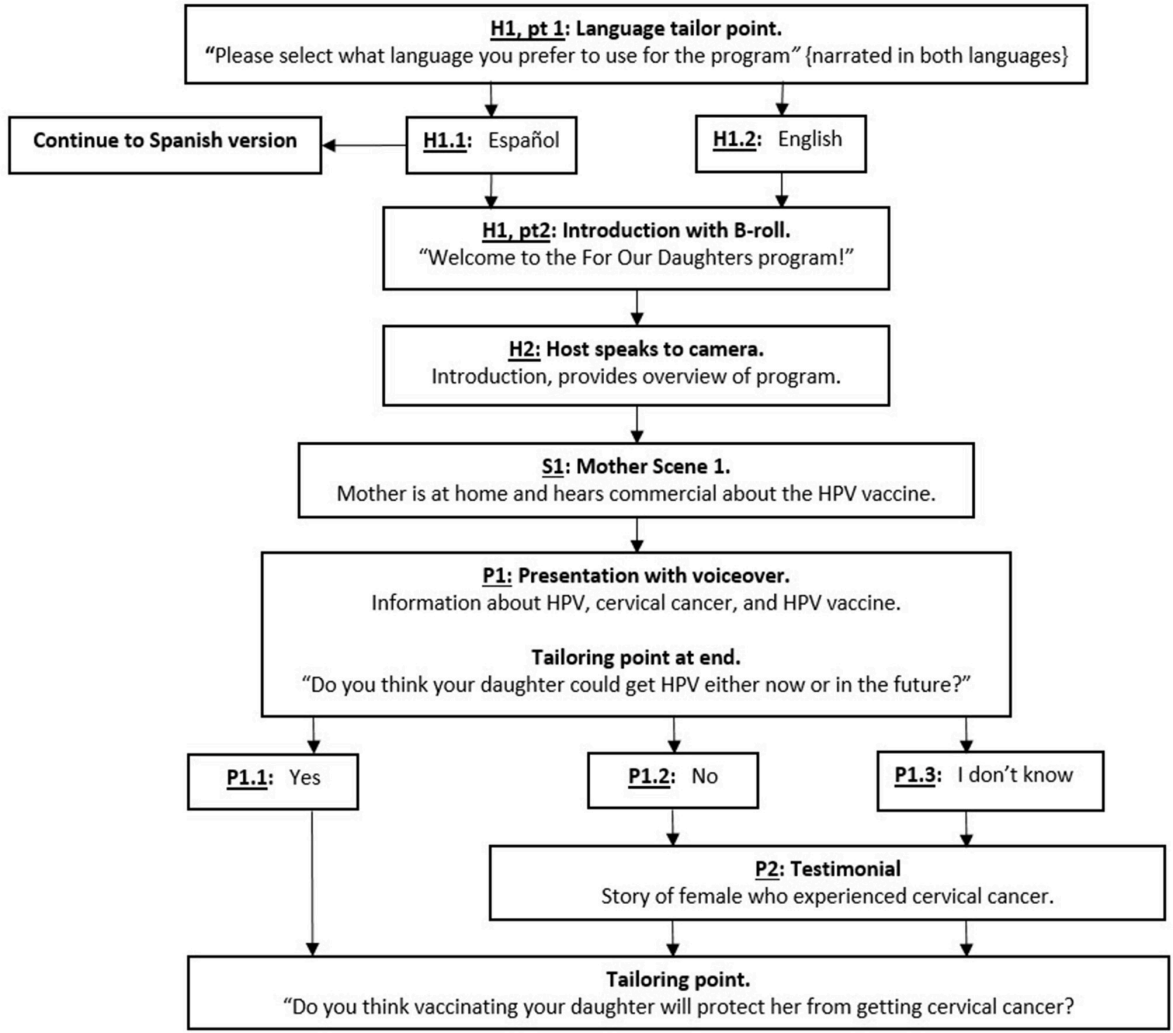
Partial TIMI flowchart for parents of Hispanic adolescent females.

Design documents shared with the TIMI design team included a flowchart, mock-up presentations, and scripts. A detailed flowchart of the TIMI included all tailoring points and detailed the placement of all video vignettes (Figure 3). Mock-ups of presentations with voiceover were created for TIMI graphic designers. Presentations included illustrations, such as abnormal cells leading to cervical cancer, a presentation on HPV prevalence, and information about free vaccination programs. Scripts were in both English and Spanish. Simple, direct sentences free of jargon were used to accommodate a low-literacy population.

**Figure 4:**
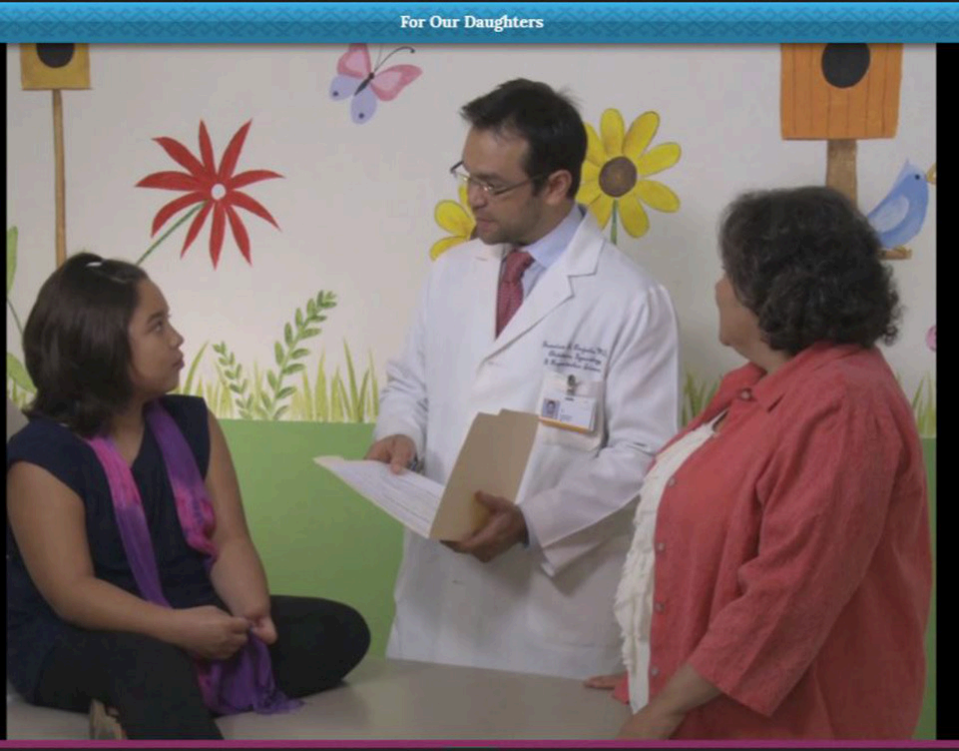
“*Por Nuestras Hijas*” screenshot.

Actors for the TIMI and *fotonovela* were bilingual Hispanic actors with experience in health-related productions. It was important to hire actors resembled our target population and to film in locations that were culturally appropriate for our target audience (e.g., community-based clinics serving predominantly low-income populations). A video production team with experience in health-related productions filmed and produced all scenes, and a graphic designer created presentations with voiceover for the TIMI. The design team received all material and created the tablet-based application (Figure 4).

**Figure 5:**
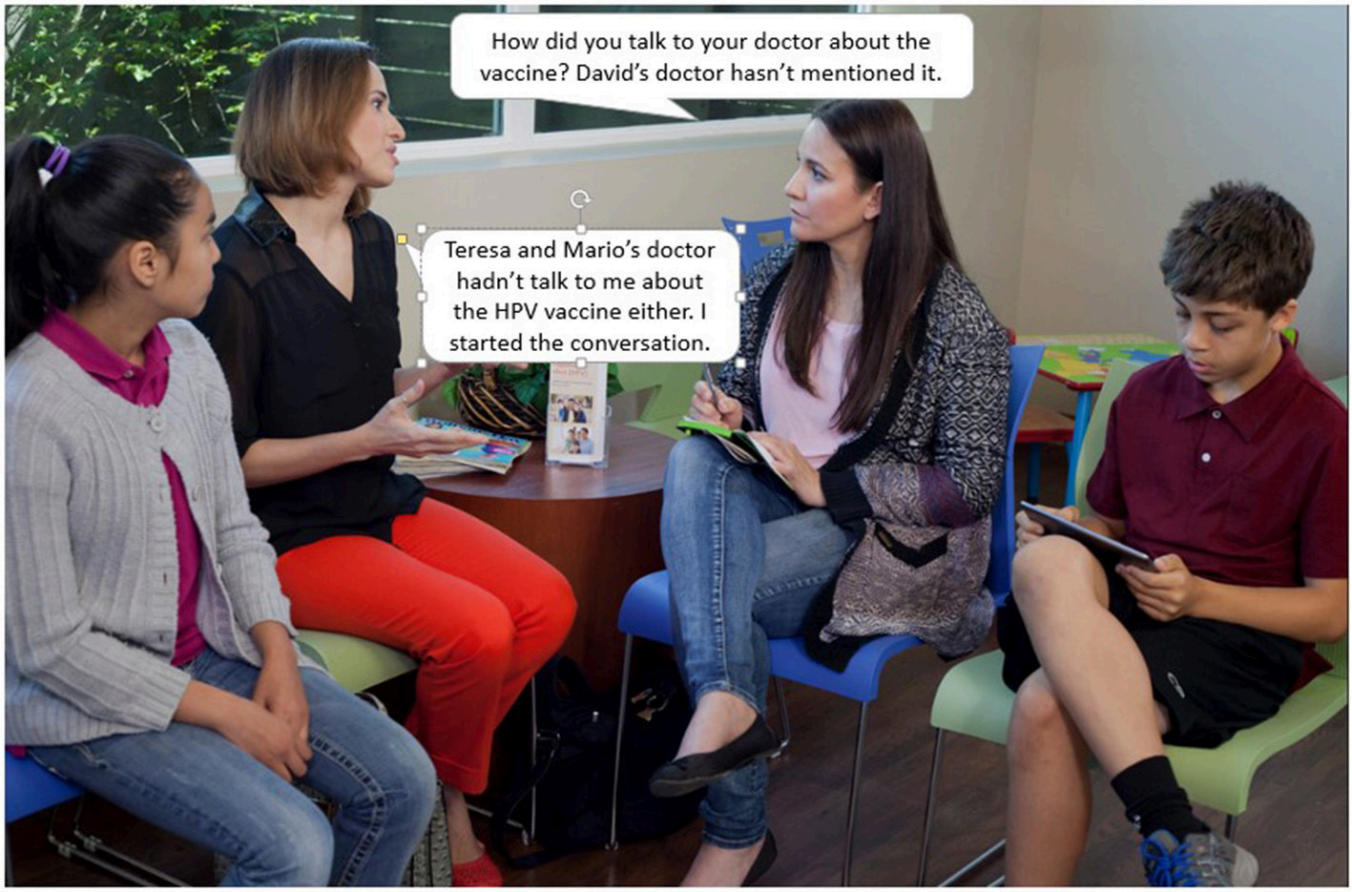
“*Por Nuestros Hijos ” fotonovela* scene.

We created a storyboard for the *fotonovela* based on scripts used in the TIMI. Similar to a comic book or illustrated novel, each page of the *fotonovela* contained short text bubbles along with a relevant image from a relevant video scene (Figure 5). In addition to the story of a mother learning about the HPV vaccine and deciding to vaccinate her daughter, the *fotonovela* contained the following content: (1) a list of resources available to help cover the cost of the vaccine; (2) information about HPV and the HPV vaccine; (3) a calendar to help parents keep track of dosing schedules; and (4) a place for mothers to write down questions about the vaccine to ask their health care provider.

The cost of developing the TIMI and *fotonovela* have previously been reported (40). As reflected in the development steps outlined here, a substantial proportion of personnel time was associated with program development. Cost analyses indicate that the personnel time cost to plan and create the *fotonovela*-based education (41%) was less than the iPad-based TIMI (67%) education intervention.

#### Step 5. Implement *Por Nuestras Hijas*

For implementation, the target behavior was “Lay health worker will deliver the educational interventions with fidelity,” and performance objectives included (1) adopting the role of lay health worker, (2) locating eligible parents, and (3) conducting sessions with parents. Determinants included knowledge, skills and self-efficacy, attitudes, perceived norms, and outcome expectations. After completing the matrix of change objectives, we identified the following theoretical methods: information, modeling, feedback, reinforcement, and active learning for use in the implementation intervention. These methods were operationalized through presentations, activities, and practice sessions during a 2-day training session at *Pro Salud*, the lay health worker program headquarters. As a part of the training, medical experts presented information about HPV, the HPV vaccine, and cervical cancer. Each presentation ended with a question and answer session. The goal was to provide the lay health workers with a working knowledge of HPV, cervical cancer, and the HPV vaccine. We also used skills training and modeling approaches to prepare the lay health workers for their role in educating parents. Lay health workers learned how to use the program materials and how to respond to participant questions or concerns through both didactic presentations and facilitated practice session. Lay health workers took turns administering the program materials to build skills and self-efficacy. During this time, research staff circulated around the room to observe, assist, and offer feedback and reinforcement. Additional training topics pertained to evaluation efforts (see IM Step 6).

#### Step 6. Evaluate *Por Nuestras Hijas*

To assess content validity, we created a table mapping all program components back to change objectives and determinants (Table 3). The table illustrates the expected mechanisms of change. For example, in IM Step 1 we identified self-efficacy as a modifiable factor associated with behavior change to increase HPV vaccination. Parents needed increased self-efficacy to discuss the HPV vaccine with providers. In order to increase self-efficacy and to address each change objective associated with the determinant, the program included scenes with a mother modeling the behavior. As part of the content analysis, we went back to the table to ensure all appropriate change objectives were included in that scene.

**Table 3:** Determinants and change objectives mapped to methods and practical applications.

**Determinant**	**Targeted change objectives**	**Method**	**Practical application**	**Program component**
Knowledge	(K)1.A. Recognize that there is an HPV vaccine	Modeling	Scene: Doctor discusses HPV vaccine with mother	Interactive multimedia/*Fotonovela*
	(K)1.B. Recognize that daughter is eligible to receive the HPV vaccine	Modeling	Scene: Doctor discusses HPV vaccine eligibility with mother	Interactive multimedia/*Fotonovela*
	(K)1.C. Understand the purpose of the HPV vaccine	Modeling	Scene: Doctor describes HPV vaccine as cancer prevention	Interactive multimedia/*Fotonovela*
	(K)1.D. Understand the link between HPV and cervical cancer	Education and counseling	Scene: Doctor describes link between HPV and HPV diseases	Interactive multimedia/*Fotonovela*
Attitude	(A)1.A. Believe that the HPV vaccine is important for their daughters	Modeling	Testimonials: Mothers discuss vaccinating daughter	Interactive multimedia/*Fotonovela*
	(A)1.B. Believe the HPV vaccine to be safe for their daughters	Modeling; Education and counseling	Scene: Doctor describes HPV vaccine as safe and effective	Interactive multimedia/*Fotonovela*
	(A)1.C. Believe that the HPV infection can have serious consequences	Modeling	Testimonials: Cervical cancer survivors describe experiences	Interactive multimedia/*Fotonovela*
Self-efficacy	(SE)2.A. Feel confident that they can initiate discussion with doctor even if HPV vaccine is not mentioned	Modeling	Scene: Mother has list of questions and asks doctor about HPV vaccine	Interactive multimedia/*Fotonovela*

We recruited 1,398 parents of adolescent females in participating clinics as part of the group randomized controlled trial to assess the effectiveness of the two interventions. Trained, bilingual data collectors approached parents in clinic waiting rooms, assessed eligibility, and obtained informed consent from those agreeing to participate. Data collectors administered baseline surveys to all participants and lay health workers administered the interventions to those in the *fotonovela* and TIMI study arms. We conducted follow up surveys and accessed patient vaccination records to assess vaccination status at 6 months after baseline. Results are forthcoming. During program development and implementation, we also assessed the cost-effectiveness of developing and implementing the interventions (40, 41). Finally, a sequential, multiple assignment, randomized trial to assess the effectiveness of *Por Nuestros Hijos* (described below) is currently underway.

### *Por Nuestros Hijos* program adaptation

#### Step 1. Develop a logic model of the problem

During the literature review, thirty-two relevant articles were identified after screening 1,032 title and abstracts. Similar to female adolescents, determinants for vaccinating adolescent males included parental knowledge, self-efficacy, skills, perceived benefits of the HPV vaccine, perceived susceptibility to HPV, perceived social norms, outcome expectations, and HPV vaccination attitudes. Parental intentions to vaccinate their sons were higher if they had daughters who were previously vaccinated against HPV (42, 43). Further, parents of males often believed their adolescent sons may soon become sexually active, and they believed vaccinating their sons could prevent the transmission of HPV to their female partners in the future (43–45). This finding was different from parents of females who perceived their daughters to be too young for the vaccine and not at risk for HPV because they did not perceive their daughters to be sexually active in the near future.

Focus groups and interviews revealed knowledge gaps about HPV-related cancers among males. Some parents were also unaware the HPV vaccine was available for males. Other knowledge gaps included parents confusing HPV with the herpes simplex virus (HSV) and parents now knowing condoms do not provide complete protection against HPV. Finally, parents reported receiving conflicting information about HPV from multiple sources. These results informed new content included in the adapted intervention.

#### Step 2. identify program outcomes and objectives—logic model of change

The adapted program focused on a new outcome (HPV vaccination of sons). However, the target population, Hispanic parents, and the desired behavior, HPV vaccination, remained the same. Therefore, performance objectives were unchanged, and the matrix of change included most of the same change objectives. Minor changes included updating the knowledge change objectives to include male cancers and genital warts and removing change objectives regarding concerns about sexual disinhibition. For parents of daughters, we originally included content that assured parents daughters were not more likely to initiate sex at a younger age or to engage in risky sexual behaviors after vaccination. For parents of sons, we did not emphasize this point since it was not an identified barrier in the literature review or focus groups and interviews.

#### Step 3. design *Por Nuestros Hijos*

Our original theme and program title *Por Nuestras Hijas* (For Our Daughters) was changed to *Por Nuestros Hijos* (For Our Children) to reflect that the program now contained information for parents of daughters and sons. The adapted program included the original story of a mother of a daughter and included a new story following a mother gathering information and deciding to vaccinate her son. The adapted program used the same program components, methods, strategies, and practical applications with one addition. Text message reminders, or cues to action, were added as a during *Por Nuestros Hijos* (For Our Children) to target HPV vaccine series completion behaviors.

#### Step 4. produce *Por Nuestros Hijos*

To adapt the TIMI, we modified the original flowchart to include gender as an additional tailoring point. The flowchart depicted the two-arm program with one arm tailored for parents with daughters and one arm tailored for parents with sons. A third arm for parents with both daughters and sons is currently under development. We also revised the original scripts and multimedia presentations to provide information relevant to HPV-related cancers in males. As before, the program includes culturally appropriate actors. We followed the same process as in the development phase to produce the *fotonovela* aimed at parents of Hispanic males. Language in text message reminders to increase series completion were based on our previous work regarding linguistic agency and HPV vaccination intentions (31).

#### Step 5. Implement *Por Nuestros Hijos*

Our implementation plan for delivering *Por Nuestros Hijos* (For Our Children) did not change from *Por Nuestras Hijas* (For Our Daughters). Lay health workers continued to implement the program. The two primary adaptations to training content included: (1) providing information about HPV-related cancers affecting males, and (2) describing the updated ACIP recommendations to include HPV vaccination for adolescent males.

## Discussion

We used Intervention Mapping (IM) to systematically develop and adapt two interventions for parents of Hispanic adolescents to increase HPV vaccine uptake. We first followed IM to develop *Por Nuestras Hijas* (For Our Daughters), an intervention targeting parents of Hispanic females. The process relied on evidence and theory to drive development of the logic model of the problem, program outcomes and objectives, program design, program production, development of an implementation intervention, and evaluation. As guidelines changed to include HPV vaccination for males (46), we used IM to assess and adapt the original program to the needs of parents of Hispanic males. We repeated IM Steps 1–6 systematically assessing the original intervention and identifying key elements that needed adapting.

Our program adapted for Hispanic parents of adolescent males required minimal adaptations. IM can guide program planners in making adaptations that are more significant by systematically guiding decision-making throughout the process. This is helpful as program planners identify evidence-based interventions developed in one context and adapt them for another (23). Changes may include a new priority population, a different setting, or a new health behavior. IM then provides the framework to assess and adapt the building blocks of each intervention to determine the level of adaptation needed.

*Por Nuestros Hijos* required mostly surface-adaptations since the behavioral outcome, determinants, and performance objectives were similar to the original program. Surface adaptations are smaller adaptations that tailor a program to a new audience, but do not necessarily add new program components or address new determinants. In *Por Nuestros Hijos*, example surface adaptations included changing characters to include males, altering scripts to include HPV-related cancers related to males, and updating the HPV guidelines to include males. Some program planners may follow IM and find that deeper adaptations, or more extensive changes to the original program, are needed to adapt an existing program for a new population. For example, IM Step 1 may identify a new determinant relevant to the new target population. This would then add to the matrix of change objectives. Planners would then identify new methods to target that determinant, new practical applications, and new intervention materials addressing the new change objectives—activities associated with more extensive adaptations to the original program.

Further, IM assists program planners in identifying the most salient methods, applications, or program components critical to program effectiveness. For example, in *Por Nuestras Hijas* (For Our Daughters), we used modeling to address parental self-efficacy in asking the provider questions, requesting the vaccine, and scheduling subsequent doses. We also used modeling to address parental outcome expectations. In this way, and based on the IM logic model of change, modeling was a critical method for creating parental behavior change. Modeling was the mechanism through which we expected to increase self-efficacy, to create positive outcome expectations, and to ultimately increase vaccination behaviors. In adapting the program, we retained modeling as a method since determinants were identical, and we adapted the original scripts to include language relevant to parents of males to operationalize the method.

Interventions targeting multiple factors associated with a health behavior are critical to behavior change, and interventions specifically targeting HPV vaccination sometimes fail to address more than one factor associated with parental decision-making. Interventions to increase HPV vaccination among adolescents often provide only educational information or cues to action (e.g., reminder letters, text messages) for parents (47). Educational interventions and materials, including those produced by the CDC, aim to increase parental knowledge about HPV, HPV-related cancers, and the HPV vaccine (48). While it is important to increase parental knowledge, focusing solely on this factor may not be enough to change behavior, particularly for unmotivated parents (49). Systematically identifying and addressing multiple factors associated with HPV vaccination, such as attitudes, beliefs, or self-efficacy, allows intervention planners to move beyond simply providing information or cues to action to create behavior change.

The steps that guided program development and adaptation are applicable to a broad group of researchers, program planners, and public health professionals. IM was specifically designed for use in public health settings, not just academic research settings, providing public health practitioners the tools and resources to develop theory- and evidence-based interventions specific to their context (11). IM provides a systematic process, appropriate for any population or behavior of interest, that result in interventions targeting determinants specific to the population and behavior. Public health practitioners and health educators in health departments and hospital settings (50, 51), workplace settings (52), and community organizations (23) have successfully developed theory- and evidence-based interventions using IM. As exemplified by *Por Nuestras Hijas* (For Our Daughters) and *Por Nuestros Hijos* (For Our Children), IM provides a user-friendly, iterative stepped approach to facilitate development and adaptation guiding researchers and public health practitioners every step of the way.

Future studies should evaluate the *Por Nuestras Hijas* (For Our Daughters) and *Por Nuestros Hijos* (For Our Children) interventions to assess their efficacy in increasing HPV vaccination in Hispanic adolescents and in changing psychosocial determinants. These studies will provide important information regarding the effect the interventions on the knowledge, beliefs and attitudes that influence HPV vaccination along with information about the efficacy of the two program components. If the interventions are effective in increasing vaccination, they could be adapted for other populations using the process we outline in this paper.

There are limitations to our development and adaptation process using IM. Participants in the focus groups and interviews self-selected to be a part of our needs assessment work. Their attitudes toward the HPV vaccine may differ from those of other parents who did not participate. We therefore may not have captured all determinants relevant to our target population. While we conducted systematic reviews of the literature to also identify determinants, the qualitative nature of the needs assessment process may limit generalizability. Additionally, the program is limited in reach since participants were low income and underinsured Hispanic parents in an urban setting. The programs may not be effective among Hispanics in other settings, and messages may not resonate for other race/ethnicity populations. Further, while our team had resources to train and implement the intervention using lay health workers, clinics and other organizations utilizing this program in the future may not be able to employ this implementation strategy. This potentially limits program reach. Similarly, clinics or other organizations may not have capacity to implement text message reminders to target HPV vaccine series completion. Our development and adaptation processes and outcomes did not account for these differences in resources for future program adopters.

## Ethics statement

This study was carried out in accordance with the recommendations of UTHealth Science Center at Houston with written informed consent from all subjects. All subjects gave written informed consent in accordance with the Declaration of Helsinki. The protocol was approved by the University of Texas Health Science Center at Houston Institutional Review Board.

## Author contributions

MF, SV, LS: Study and manuscript conceptualization; SR: Background, methods, results, discussion; AR, LS, and MF: Contributed to background, methods, results, discussion; DL: Contributed to methods.

### Conflict of interest statement

The authors declare that the research was conducted in the absence of any commercial or financial relationships that could be construed as a potential conflict of interest.
